# Can the theoretical domains framework account for the implementation of clinical quality interventions?

**DOI:** 10.1186/1472-6963-13-530

**Published:** 2013-12-21

**Authors:** Wendy Lipworth, Natalie Taylor, Jeffrey Braithwaite

**Affiliations:** 1 Centre for Clinical Governance Research, Australian Institute of Health Innovation, University of New South Wales, AGSM Building (L1), Randwick, NSW 2052, Australia; 2 Bradford Institute for Health Research, Bradford Royal Infirmary, Duckworth Lane, Bradford, Yorkshire BD9 6RJ, England

**Keywords:** Theoretical domains framework, Clinical quality, Health service management, Behaviour change, Qualitative research, Qualitative synthesis

## Abstract

**Background:**

The health care quality improvement movement is a complex enterprise. Implementing clinical quality initiatives requires attitude and behaviour change on the part of clinicians, but this has proven to be difficult. In an attempt to solve this kind of behavioural challenge, the theoretical domains framework (TDF) has been developed. The TDF consists of 14 domains from psychological and organisational theory said to influence behaviour change. No systematic research has been conducted into the ways in which clinical quality initiatives map on to the domains of the framework. We therefore conducted a qualitative mapping experiment to determine to what extent, and in what ways, the TDF is relevant to the implementation of clinical quality interventions.

**Methods:**

We conducted a thematic synthesis of the qualitative literature exploring clinicians’ perceptions of various clinical quality interventions. We analysed and synthesised 50 studies in total, in five domains of clinical quality interventions: clinical quality interventions in general, structural interventions, audit-type interventions, interventions aimed at making practice more evidence-based, and risk management interventions. Data were analysed thematically, followed by synthesis of these themes into categories and concepts, which were then mapped to the domains of the TDF.

**Results:**

Our results suggest that the TDF is highly relevant to the implementation of clinical quality interventions. It can be used to map most, if not all, of the attitudinal and behavioural barriers and facilitators of uptake of clinical quality interventions. Each of these 14 domains appeared to be relevant to many different types of clinical quality interventions. One possible additional domain might relate to perceived trustworthiness of those instituting clinical quality interventions.

**Conclusions:**

The TDF can be usefully applied to a wide range of clinical quality interventions. Because all 14 of the domains emerged as relevant, and we did not identify any obvious differences between different kinds of clinical quality interventions, our findings support an initially broad approach to identifying barriers and facilitators, followed by a “drilling down” to what is most contextually salient. In future, it may be possible to establish a model of clinical quality policy implementation using the TDF.

## Background

Health care policymakers, managers, clinicians and educators have now spent several decades attempting to improve the quality of health care through the application of a wide range of clinical quality interventions. Collectively, these refer to interventions aimed at encouraging clinical services to be safer, and more appropriate, efficient, effective, accessible and consumer-focused. Clinical quality interventions include large-scale changes to organizational structures such as new networks among doctors; managerial processes for monitoring clinical work and the concomitant IT systems; incentive payments; and systems for analysing the root causes of harm. They also include smaller-scale activities focused on (for example) the definition and management of clinical indicators; the conduct of clinical audits and practice accreditation; the development and promotion of clinical practice guidelines; and the institution of continuous quality improvement, risk management and incident management systems. Despite considerable efforts, the changes promoted by those concerned about the quality of health care have not been applied consistently, and progress in improving the quality of care has been painfully slow [1].

Two possible explanations for this state of affairs can be adduced. One is that clinical quality interventions are *themselves ineffective* and not supported by a strong evidence base. A second is that these interventions have not been *implemented effectively*[2]. We know that some clinical quality interventions are not supported by strong evidence, while uptake and spread of others is poor even where evidence of benefit of the intervention is clear, as in the use of rapid response systems [3,4], checklists [5,6], or interventions to improve hand hygiene [7,8] and central venous line safety [9]. It is therefore likely that both accounts play a role at various times.

There are in turn two possible overarching explanations that encompass both possibilities. The first is that there are structural barriers that prevent the uptake of even the most evidence-based interventions. The second, which will be the focus of this article, is that we do not understand enough about the factors that make it more or less likely that clinicians will implement clinical quality interventions. Typically, the methods for facilitating uptake by clinicians of clinical quality interventions are developed intuitively and rely on proponents educating, persuading, or reminding people to change their behavior [10]. In addition, theories underpinning the implementation of clinical quality interventions, which require healthcare professionals to change their behavior, are seldom explicated [11]. This makes it difficult to identify the specific components of interventions that have been used, and of those, which were effective. This, in turn, makes replication of successful interventions problematic. What health behavior change theories offer is a mechanism to understand how both internal (e.g., motivation, self-efficacy), and external (e.g., environmental context, resources), barriers might affect implementation, and a means by which tailored and theoretically underpinned interventions can be designed to address those barriers.

Why might it be the case that people are so poor at mobilizing theory to support their interventions? It could be that change agents are unaware of behaviour change theory, and neglect to draw upon any conceptual perspective. Or it could be that those tasked with designing and implementing behaviour change interventions find it difficult to choose from the abundance of health behaviour theories, such as the health belief model [12], the theory of reasoned action [13,14], organisational culture change models [15-18], the theory of planned behaviour [19,20] or social cognitive theory [21]. And even if they did seek to apply a particular theory, change agents may find that their selected theory is insufficiently broad to account for all of the factors that motivate or thwart behavior change.

In an attempt to solve this kind of behaviour change problem, the theoretical domains framework (TDF) has been designed to apply in a variety of settings. The TDF consists of a set of conceptual determinants and associated constructs from psychological and organisational theory that putatively influence behaviour and stimulate behaviour change [22]. The framework was established to be used by anyone, from any discipline, who needs to accurately identify barriers and levers to behaviour change, and design interventions with sufficient theoretical richness to address them [23]. The TDF has recently been updated [24] and consists of 14 domains (knowledge, skills, social and professional role and identity, beliefs about capabilities, optimism, beliefs about consequences, reinforcement, intention, goals, memory, attention and decision processes, environmental context and resources, social influences, emotion, and action planning).

**Figure 1:**
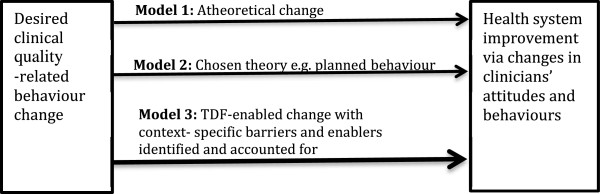
Three models of clinical behaviour change.

Three possible models of change emerge from the discussion so far: behavior change that is not based on any theoretical framework; behavior change that is motivated by a theory focused on one specific dimension of behavior change; and behavior change that is based on the TDF. These are presented in Figure 1.

The TDF has been applied in clinical contexts to understand the determinants of behaviour change such as hand hygiene [25] and transfusion practice [26]. However, no systematic research has been conducted into the extent to which, and ways in which clinical quality interventions more generally map to the various domains of the TDF. Thus those who might want to use the TDF (model 3 in Figure 1) to guide clinical quality interventions have no way of determining *a priori* how or in what ways it will be useful.

### Aims and research questions

With this background in mind, we set out to conduct a mapping experiment to address the following questions:

1) Which (if any) domains of the TDF are relevant to the implementation of clinical quality interventions?

2) How do these domains express themselves in the context of various types of clinical quality interventions?

3) Which (if any) domains of the TDF are not relevant to the implementation of clinical quality interventions?

4) What (if any) barriers to, or facilitators of, clinical quality interventions are not accounted for by the TDF?

We attempted to answer these questions by analysing the attitudes of clinicians towards clinical quality interventions, because it is their beliefs and perspectives, and in turn their behaviour, that are ultimately the target of clinical quality initiatives. We chose a qualitative method to appraising such attitudes because this allowed for:

1) A detailed and nuanced assessment of the relevance of the TDF to clinical quality interventions,

2) An analysis of both implicit and explicit responses and attitudes to clinical quality interventions (i.e. that allowed us to identify barriers and facilitators that were not directly expressed as such), and

3) Findings to emerge from the data, so that we could determine which (if any) domains of the TDF were most salient in the clinical quality context without “forcing” the data.

### Rationale for a synthesis of existing qualitative research

There is already a large body of qualitative literature exploring clinicians’ perceptions of, attitudes towards, and responses to various clinical quality initiatives. This literature has not been synthesised. Rather than conduct costly primary qualitative research into clinicians’ experiences, understandings and attitudes, we therefore chose to utilise existing studies.

There is precedent for such an approach, and it is now generally accepted that syntheses of qualitative research, like meta-analyses of quantitative studies, can provide new insights and inform clinical and public health practice [27-30] as well as the work of health service managers and policy-makers [2]. It has been recognised that research syntheses do not need to be limited to questions about the effectiveness of particular interventions but can also focus on “broader contextual factors” ( [2] p59) such as behaviour change.

## Methods

### Conceptual framework

Methods for synthesizing qualitative research are currently undergoing rapid development [29]. Our method of qualitative synthesis was based on Thomas and Harden’s description of “thematic synthesis” [31]. Like methods such as Noblit and Hare’s [32] “meta-ethnography” [28,32] and Sandelowski and Barroso’s [33] approach to “meta-synthesis,” [33] thematic synthesis involves identifying key themes in published studies, and then going beyond the studies to identify similarities and conflicts, and to offer novel interpretations, “lines of argument,” or “third-order” concepts not found in any single study [32,34].

Although qualitative synthesis is in some ways the qualitative analogue to meta-analysis in quantitative studies, there are also a number of key differences between quantitative and qualitative syntheses. The primary difference is that meta-analyses (and quantitative systematic reviews more generally) rely on identifying and synthesizing *all* key sources of data, while qualitative syntheses—like qualitative research more generally—aim to analyse as many sources as are necessary to achieve *thematic saturation,* where thematic saturation refers to the point at which no new themes are emerging from the data. For this reason, comprehensive and unbiased literature searching is not as important in qualitative syntheses as it is in quantitative systematic reviews and meta-analyses [33].

### Identification of articles for review

We initially searched Google Scholar using the phrase “qualitative clinical governance”^a^. As proposed by Sandelowski and Barroso [33], our aim was to recall as many articles as possible; that is, we sought sensitivity more than specificity. We therefore deliberately chose a broad and nonspecific search engine—Google Scholar, and kept our search term broad so as to avoid missing important articles. This initial search identified hundreds of potentially relevant studies. We subsequently included studies if they met the following criteria:

1) The article was relevant to our research questions, i.e., it explored clinicians’ views and experiences of clinical quality interventions. The most common exclusions according to this criterion were articles on the effectiveness of particular clinical quality interventions, or articles exploring the attitudes of managers rather than clinicians,

2) The data collection and analysis methods were reported as qualitative by the authors, and

3) The article was published in English in a peer-reviewed journal.

No date limits were set as we thought that old studies might remain relevant. We deliberately did not limit our field to studies of clinical quality interventions that have been shown to work—i.e. are evidence based, because we were interested in whether and how beliefs about evidence (or lack thereof) might affect uptake of interventions.

In order to ensure that our analysis was sufficiently broad, we divided articles into five categories and ensured that all categories were represented (Additional file 1: Table S1):

1) Perceptions of clinical quality interventions in general (“general”)

2) Perceptions of changes to organizational structures aimed at quality assurance and improvement (“structural”)

3) Perceptions of quality assurance activities such as target setting and auditing, (“audit”)

4) Perceptions of activities aimed at making practice more evidence-based (“EBM”), and

5) Perceptions of continuous quality improvement, risk and incident management activities (“risk management”).

It is important in this kind of qualitative research to achieve as much variation as possible so that important themes are not missed, We therefore developed these categories based on our broad collective knowledge of the types of interventions that concern those with an interest in clinical quality. Our goal was not to develop a formal taxonomy of clinical quality interventions, but rather to ensure that we did not skew our analysis towards only one kind of intervention. For the same reason, we included studies that focused on attitudes towards specific clinical quality interventions, as well as studies that focused on attitudes towards clinical quality interventions in general.

In order to ensure that no major categories of clinical quality interventions had been missed, we also searched for articles published in the past 5 years in Web of Knowledge, Medline, PsycINFO, EMBASE and CINAHL using a number of search terms relevant to clinical quality, in combination with “qualitative,” and the names of qualitative methodologies (such as grounded theory, ethnography, case study, discourse, action research, and narrative) and data collection techniques (such as interview and focus group). No additional categories of clinical quality intervention were identified.

### Appraisal

We found it difficult to exclude studies on the basis of methodological quality because of the frequent lack of detail in reporting methods and methodology, and the well-recognized epistemological challenges of critically comparing different qualitative methodologies [29]. Given that our aims were to find maximum variability and to usefully interpret the literature through the TDF rather than identify the “best” publications on the topic, or conduct a complete systematic review, we decided, following Thomas and Harden [31] and Atkins et al. [27], to err on the side of inclusion, and to judge quality on the basis of conceptual contribution as much as methodological rigor. Our “pragmatic” approach to the selection and appraisal of articles is also consistent with the recognized challenges of synthesizing research for managers and policymakers, as distinct, for example, from the highly formulaic approaches to synthesizing quantitative studies of clinical effectiveness [2].

### Assessing “saturation”

This initial search, together with secondary searching of papers referenced in other studies, generated a very large number of relevant results (over 150 relevant studies) and we were able to reach thematic saturation comfortably without needing to search other databases. For the reasons described above, this is an appropriate approach in conducting qualitative synthesis, where thematic saturation is the primary determinant of when one can “safely” cease data collection. In retrospect, thematic saturation was reached after we had analysed approximately 25 articles (5 in each category). We analysed 10 articles in each category, giving a total of 50 articles. The 10 articles in each category were selected purposively to ensure we obtained as much variety as possible within each category. While this was a relatively large number of articles compared to some thematic analyses, we have found in the past that large syntheses are both feasible and productive [35-37].

### Data analysis

In order to develop third order concepts, we drew both on Morse’s outline of the cognitive basis of qualitative research [38] and on Charmaz’s outline of data analysis in grounded theory [39]. This procedure involved initial line-by-line coding, synthesizing codes into categories until no new codes could be developed from the data, focused coding using these categories, and abstracting into third order concepts. A coding tree was generated. Throughout the data analysis, a process of constant comparison was employed. Existing codes, categories, and concepts were refined, enriched, and reorganized as new codes; and categories and concepts were developed or as similarities and differences were recognised. Enough material was analysed to ensure that categories were saturated and all analytic third order concepts were fully described and well understood.

Importantly, we did not enter into the research process with the assumption that the domains of the TDF would account for the concepts we developed. This only emerged after we had analysed the interviews and the categories and concepts were developed inductively from the data. In other words, the research was conducted in two phases: an initial “open” phase, in which any themes, categories and concepts were allowed to emerge from the data, followed by a “focused” phase, in which we then mapped the concepts that had emerged to the domains of the TDF. In this way, we were able to avoid “forcing” the data to cohere with the domains of the TDF.

We therefore approached analysis of the manuscripts inductively with the following broad research question:

1) What do clinicians explicitly believe to be the key barriers to, and facilitators of, clinical quality interventions?

2) What other barriers and facilitators are implicit in clinicians’ discourse?

We then asked ourselves where, if anywhere, these barriers and facilitators would fit into the TDF (i.e. we asked the more focused research questions listed previously).

WL conducted the initial “open” analysis from the original articles. WL and NT re-categorized these independently according to the domains of the TDF and, together with JB, reached an agreement as to where (if anywhere) each code belonged.

## Results

### Included studies

We included 10 studies in each of the five broad categories of clinical quality interventions. Additional file 1: Table S1 describes each study in terms of:

1) The clinical group/s involved

2) The country in which the study was conducted

3) The number of research participants

4) The type of clinical quality initiative being considered

5) The method of qualitative data collection.

### Domains of the TDF as they are represented in clinical quality interventions

Here we capture how each of the domains of the TDF emerged in the qualitative studies we analysed. The references to analysed studies are given in the form [Citation number_year, category of clinical quality intervention] to facilitate finding their details in Additional file 1: Table S1.

#### Knowledge

In the TDF, “knowledge” refers to “an awareness of the existence of something.” This is believed to be important because a person’s perceived awareness of the scientific rationale, procedure(s), and task environment associated with a desired behaviour is likely to affect whether a person decides to implement it.

Knowledge emerged as a salient domain in the context of all five of our categories of clinical quality interventions. High levels of knowledge were seen to be a facilitator of change. For example, primary care practitioners believed that the uptake of clinical practice guidelines would be facilitated by their being informed about which guidelines to follow and where to find them ( [40]; EBM). In contrast, low levels of knowledge were seen to be a barrier. For example, Primary Care Organisation (PCO) leads, discussing primary care clinical quality policy in general, argued that a major barrier to implementation was the lack of guidelines on non-clinical aspects of the quality framework ( [41]; General).

#### Skills

According to the TDF, “skills” refer to “an ability or proficiency acquired through practice”. Skills are thought to be important determinants of behavior change because a person’s perceived sense of their own competence in performing a desired behaviour is likely to affect whether or not they are willing and able to implement it. The provision and quality of training for skill development, opportunities to practice, and opportunities to gain an understanding of an existing skill set through assessment are also likely to influence performance of the desired behaviour.

As with knowledge, the sense that one was adequately skilled was a facilitator of behavior change, while perceived lack of skill was a barrier to change. For example, hospital doctors discussing medical audit thought that a major barrier was that not all doctors are adequately trained in audit ( [42]; Audit), while senior primary care clinicians, discussing the cultural changes needed to implement clinical quality interventions in general practice, noted that a barrier was lack of necessary skill in implementing these interventions ( [43]; General).

The perception that an intervention was “user friendly” was closely related to the perception that one had the skills to perform it. In one study of allied health and management personnel’s attitudes to trans-disciplinary teamwork, for example, informal communication was seen to be a natural and fluid process for most clinicians and one that was approved of and accepted by all clinicians that were interviewed ( [44]; Structural). This was seen to facilitate implementation. In contrast, in relation to a patient safety incident management system, a significant proportion of hospital clinicians noted that they found the system confusing or difficult to use and that this impeded implementation ( [45]; Risk).

#### Beliefs about capabilities

In regard to “beliefs about capabilities” the TDF refers to “acceptance of the truth, reality, or validity about an ability, talent, or facility that a person can put into constructive use”. Beliefs about capabilities are relevant because the level of confidence an individual possesses about their ability to perform a particular behaviour is likely to affect whether or not they implement it.

Unlike knowledge and skills, where high levels were always facilitative, high levels of perceived self-efficacy could be both a barrier and a facilitator of behavior change. On the one hand, belief in one’s capacity to implement an intervention could be facilitator of uptake of the intervention, while lack of belief in one’s capacity was a barrier. For example, primary care doctors expressed concern about their capabilities to apply clinical practice guidelines for the management of chronic diseases ( [46]; EBM), while a perceived facilitator of use of an electronic prescribing system in primary care was that primary care doctors and other primary care staff who had a solid background in computer use had confidence in their abilities ( [47]; Risk).

On the other hand, beliefs in one’s own capabilities to perform well *without* the clinical quality intervention could, somewhat paradoxically, be a barrier to uptake of the intervention. For example, reflecting on the use of decision aid software programs in tele-nursing, nurses believed that they were competent enough to handle the common practice without using software programs. In this context, belief in one’s own capabilities was a barrier to implementation of a clinical quality intervention ( [48]; EBM).

#### Beliefs about consequences

In the TDF, “beliefs about consequences” refer to an “acceptance of the truth, reality, or validity about the outcomes of a behaviour in a given situation.” The beliefs a person holds about the outcomes of particular behavior will affect whether or not they decide to comply.

Beliefs about consequences fell into two categories in our data. First, there were beliefs about whether a needed intervention would be effective in achieving its clinical or organisational aims. Belief in effectiveness was a facilitator, whereas belief in ineffectiveness was a barrier. For example, hospital and primary care clinicians reflecting on their use of clinical practice guidelines for chronic obstructive pulmonary disease acknowledged that they were much more likely to adhere to a recommendation to communicate with patients about smoking cessation than they were to follow a recommendation to educate patients about medication self-management. This was because of their stronger belief in the health benefits of smoking cessation for their patients ( [49]; EBM).

Second, were beliefs about whether an intervention would cause any predictable or unexpected clinical or organisational harm. For example, primary care doctors indicated that public information about performance indicators could work negatively. They were greatly concerned that patients could misconstrue, misinterpret, or not have enough medical knowledge to assess published information on physicians’ performance. This was seen as a barrier to implementation ( [50]; Audit).

Of relevance to beliefs about consequences, clinicians often referred to the internal and external validity of the clinical quality instrument in question. Not surprisingly, interventions with high levels of validity were expected to work, and be relevant to specific contexts, and were more likely to be taken up than interventions that were perceived to be lacking in internal or external validity. For example, primary care clinicians argued that performance indicators were more likely to be used if clinicians saw these indicators as being “evidence-based” ( [51]; Audit), while clinical practice guidelines were less likely to be used if primary care doctors saw the evidence upon which guidelines were based as being uncertain, inconsistent, limited and/or complex ( [46]; EBM).

In this regard it is noteworthy that while clinicians were often concerned about the internal and external validity of specific clinical quality *instruments*, they did not often comment of the presence or absence of a *research* evidence base for the general *type of intervention* in question. For example, while clinicians might have been concerned about whether a particular performance indicator or clinical practice guideline was based on solid and contextually relevant clinical evidence, they did not express much interest in whether indicators or guidelines in general had been demonstrated through research to be effective types of interventions. A few possible exceptions to this were the finding that some clinicians (throughout the health system) believed that there is no clear evidence to suggest that clinical governance contributes to the quality improvement of clinical care ( [52];General) and, more specifically, the finding that some hospital clinicians were skeptical about applying evidence during a ward round in part because of a general lack of faith in the “evidence-based approach” to health care ( [53];EBM).

#### Social/professional role and identity

According to the TDF, “social and professional role and identity” refers to a “coherent set of behaviours and displayed personal qualities of an individual in a social or work setting”. The extent to which someone believes that a particular behaviour aligns with their social/professional identity is likely to influence whether or not they will implement it.

Uptake of interventions was facilitated by the perception that an intervention was or would be consistent with, or strengthen, a clinician’s social/professional role or identity. Threats to role and identity, on the other hand, could be barriers to uptake. For example, hospital doctors in one study felt that improving quality of health care was integral to their role. Reviewing clinical care against defined standards, monitoring and improving patient outcomes and comparing performance with peers were seen as legitimate activities, and this encouraged implementation ( [54]; General). In contrast, hospital doctors and nurses in another study expressed uncertainty as to their responsibility for completing a new drug prescribing sheets (nurses vs. doctors), and thus made them reluctant to comply ( [55]; Risk).

The perceived effect of a clinical quality intervention on clinician autonomy and leadership was an important subcategory of this domain of the TDF. For example, reflecting on an “evidence-based ward round” on a delivery suite, some hospital clinicians expressed their reservations about conducting a ward round that would allow all members of staff to question decision making by the lead clinician on the labour ward. It was argued that this reluctance might be related to the fear of loss of autonomy by various groups of clinicians on delivery suite ( [53]; EBM).

A number of clinicians also observed that an intervention was more likely to be implemented if it was consistent with the current organizational and professional “culture” (and vice versa). Senior primary care doctors noted, for example, that if clinical quality interventions are to be accepted by clinicians, there is a need to change the culture of general practice to one that is more focused on accountability, collaboration between practices, and reflective learning ( [43]; General).

#### Social influences

In the TDF schema, “social influences” refer to “those interpersonal processes that can cause individuals to change their thoughts, feelings, or behaviours”. Factors such as pressure, encouragement, or support from others can often influence the performance of a desired behaviour.

Social influences could be both facilitators of, and barriers to, uptake of clinical quality interventions. For example, primary care clinicians argued that practices were more likely to develop plans to act on performance indicators if there was agreement amongst the team on the purpose, benefits and importance of indicators ( [51]; Audit). In contrast, members of mental health teams, reflecting on accountability in the establishment of interdisciplinary mental health teams, were concerned about lack of responsiveness on the part of the steering group charged with responding to clinicians’ concerns ( [56]; Structural).

#### Environmental context and resources

In the TDF, “environmental context and resources” refer to “circumstances of a person’s situation or environment that discourages or encourages the development of skills and abilities, independence, social competence, and adaptive behaviour”. It is held that the nature of the environment in which a person is required to perform a specific behaviour is likely to affect whether or not a person is able or willing to perform it.

Not surprisingly, a barrier to implementation was the perception that there were inadequate resources to implement an intervention, or an environment that was not conducive to change. In one instance, lack of assessors was a major concern to primary care staff being asked to implement a “Quality Team Development Programme” ( [57]; Structural). Concerns about resources were often expressed in terms of too many demands being made too quickly. For example, some primary care clinicians felt that evidence-based healthcare, and the associated practical requirements, represents one aspect of rapid and unwanted change in the workplace ( [58]; EBM). Related to this was the sense of whether the intervention was compatible with current work practices. For example, inter-professional teamwork was seen by hospital staff to be hindered by the fact of doctors being spread through hospital, and facilitated by multidisciplinary team meetings ( [59]; Structural).

#### Optimism

According to the TDF, “optimism” refers to “the confidence that things will happen for the best or that desired goals will be attained”. This argument suggests that the extent to which a person believes a goal will be achieved will affect the likelihood of them performing the behaviour(s) that will lead to that goal.

Optimism could facilitate the uptake of clinical quality interventions. For example, primary care staff were largely enthusiastic about the benefits of computing for general practice and were optimistic about the potential for computers to present guidelines in a manageable format ( [60]; EBM). In contrast, primary care doctors and practice nurses reflecting on a “Quality Team Development Programme” worried that once the assessment visit was over, QTD would be forgotten until the next visit ( [57]; Structural).

#### Emotion

For the purposes of the TDF, “emotion” refers to “a complex reaction pattern, involving experiential, behavioural, and physiological elements, by which an individual attempts to deal with a personally significant matter or event”. It is thought that negative emotions such as fear and anxiety, and positive emotions such as joy and pride, associated with a desired behaviour, are likely to affect whether or not a person decides to perform it.

The belief that one would have a positive emotional experience—such as pride, satisfaction, catharsis or enjoyment could facilitate uptake of clinical quality interventions, while the belief that the experience would be frightening, exposing, humiliating, guilt-inducing, demoralizing, confusing, or boring was a barrier. For example, reflecting on their preferences as to whether clinical quality interventions in primary care should be locally or centrally managed, many of the proponents of a local approach stated that they found the process enjoyable ( [61]; Structural). In contrast, hospital clinicians discussing the introduction of a computerized provider order entry system noted the potential for exposing knowledge deficits and increasing conflict, and concern about “computerphobia”. The implementation period was therefore recognized as a time of potential stress and errors ( [62]; Risk).

#### Reinforcement

In the TDF, “reinforcement” means “increasing the probability of a response through a dependent relationship, or contingency, between the response and a given situation”. Reinforcement is believed to be important because the perceived rewards and punishments associated with performance or non-performance of a particular behaviour are likely to affect whether or not someone decides to implement it.

Expectation of reward was seen to be a facilitator of compliance with clinical quality interventions. For example, primary care doctors noted that the benefits of participating in significant event analyses included appraisal, training practice accreditation, and gaining the RCGP Practice Accreditation Award ( [63]; Risk). On the other hand, expectations of punishment, or lack of reward, resulting from implementation of an intervention, could be a barrier. For example few primary care doctors considered that performance indicators could be used in a positive manner to enhance their clinical practice or to reward them ( [64]; Audit). Of course, the expectation that one would be punished for *not* implementing an intervention could motivate compliance. Reflecting on clinical quality interventions in general, occupational therapists noted that avoiding censure—e.g. through thorough documentation—was an important incentive for complying with these interventions ( [65]; General).

#### Intention

The TDF sees “intention” as referring to “a conscious decision to perform a behaviour or a resolve to act in a certain way” (e.g., I intend to check the vital signs of my post-surgical patients more frequently). It is held that the level of motivation a person has or commitment that they make to act in a particular way is likely to affect whether or not they do so.

Uptake of clinical quality interventions was seen to be facilitated by the strength and stability of clinicians’ intention, or readiness to change. Hospital nurses contemplating consumer participation in acute care, for example, expressed a commitment to working towards this model ( [66]; Structural). On the other hand, lack of intention or commitment could be a barrier. For instance, reflecting on the use of clinical practice guidelines for chronic disease, it was noted that primary care doctors could lack the motivation to apply evidence, irrespective of its quality ( [46]; EBM).

#### Goals

“Goals” in TDF parlance refer to “mental representations of outcomes or end states than an individual wants to achieve” (e.g., my goal is to monitor my patients every 15 minutes for the first four hours after surgery). The existence of a goal and the value placed on it in relation to a particular behaviour is likely to influence whether or not someone decides to activate that behavior.

Uptake of clinical quality interventions could be facilitated by consistency of the intervention with clinicians’ goals and priorities. In a study, occupational therapists argued that, in the face of various “accountability dilemmas”, professionals had to choose about how to enact their various obligations. This involved setting “accountability priorities” ( [65]; General). On the other hand, lack of consistency of clinical quality interventions with clinicians’ goals and priorities could be a barrier to the implementation of these interventions. For example, hospital doctors argued that they often had to prioritise urgent clinical assignments over nurses’ requests to clear up ambiguous drug prescriptions in a new drug record system ( [55]; Risk).

#### Memory, attention and decision processes

In the TDF, “memory, attention and decision processes” refer to “the ability to retain information, focus selectively on aspects of the environment, and choose between one or more alternatives”. Remembering to enact a particular behavior, or remaining focused on it, is likely to affect whether or not the behaviour is implemented.

Uptake of clinical quality interventions was seen to be easier if the intervention was designed in such a way that clinicians would pay attention to it, remember to implement it and make the necessary decision. Primary care doctors argued, for example, that persistent, non-conflicting and repeated exposure to recommendations was an important facilitator of uptake of clinical prescribing guidelines in primary care ( [67]; EBM). In contrast, if clinicians were unable to focus on an intervention, this could be a barrier to behavior change. For example, primary care doctors reflecting on clinical decision support alerts were aware of the potential for “alert fatigue,” and expressed a desire for alerts to be relevant ( [47]; Risk).

#### Behavioural regulation

In the TDF, “behavioural regulation” refers to “anything aimed at managing or changing objectively observed or measured actions”. This is held to be important because the existence of an action plan, or monitoring progress towards a behaviour, is likely to influence whether or not a behaviour is performed or an outcome is achieved.

In the articles we analysed, willingness to comply with clinical quality interventions was associated with clinicians’ ability to self-monitor, plan their actions and break habits. For example, reflecting on establishing a “Quality Team Development Programme,” clinicians from multiple groups argued that uptake could be facilitated by acknowledging the attitudes of those whose behaviour was being audited and modifying the audit process to accommodate them, and by allowing clinicians to control the process ( [68]; Audit). Of course, leaving primary care clinicians to monitor their own performance could also allow them to game it by distorting their behavior to improve their repeated performance in audits ( [69]; Audit).

#### Trustworthiness and justice

Two particularly interesting categories to emerge from our data, which did not fit easily into any single domain of the TDF, were those of “perceived justice” and the “perceived trustworthiness” of the managers and policymakers asking for behavior change. With respect to justice, some clinicians saw the demands made by those promoting clinical quality initiatives as appropriate, fair and legitimate, while others perceived the demands as being fundamentally unjust. For example, junior hospital doctors expressed resentment about the fact that, since they carry out the day to day duties of clerking and managing patients, it was primarily their work that was being monitored through audit processes ( [42]; Audit).

Perceived trustworthiness had two components: 1) perceived technical competence and objectivity and 2) perceived benevolence—the motivation to do good for others. First, some clinicians spoke of being more willing to implement interventions if they saw those involved as technically competent. For example, primary care doctors had varying perceptions of the professional status of peer reviewers who were giving feedback on significant event analyses. When it emerged that reviewers were largely ‘frontline’ primary care doctors who were trained and experienced in giving peer feedback, skeptical participants found this encouraging and reassuring ( [70]; Risk). Second, beliefs about the goodwill, or otherwise, of those promoting clinical quality interventions also featured strongly in the studies. For instance, some hospital doctors felt that they shared a common goal with management related to improving the quality of care for patients. For others, however, the hospital was also seen as prioritizing financial objectives and government performance targets over quality improvement ( [54]; General).

## Discussion

In this research we set out to determine the extent to which, and characterise the ways in which, the various domains of the TDF played out in the context of clinical quality. We did this by synthesizing the findings of 50 qualitative studies of clinicians’ perceptions and experiences of clinical quality interventions, and then organizing these findings according to the domains of the TDF.

Two aspects of our findings were striking. First, it was clear from our results that the TDF accounts to a considerable extent for the barriers to, and facilitators of, behavior change in the clinical quality context. With the exception of the findings we have classified under the headings of “perceived justice” and “perceived trustworthiness”, there were no findings in the 50 studies we analysed that could not be mapped to one or more domains of the TDF. The TDF therefore appears to provide a comprehensive account of the barriers to, and facilitators of, uptake of clinical quality interventions.

Second, it was clear that all 14 domains of the TDF are relevant to clinical behavior change as envisaged in the thinking of those promoting clinical quality. Furthermore, each of these domains appeared to be relevant to many different types of clinical quality intervention. We did not find, for example, that emotional concerns were specific to potentially humiliating audit activities, or that concerns about knowledge and skills were specific to highly technical interventions. This suggests that the TDF can be usefully applied flexibly in any clinical quality context, whether the intervention is a large structural change, an audit-related activity, an EBM-related activity, an incident management activity or an error prevention activity.

### Theoretical implications

There are implications here for theories of clinical quality implementation. It may be that what is missing from clinical quality interventions is an adequate theoretical model, and we have shown in principle that the TDF can adequately account for much behaviour change (or lack thereof) in the clinical setting. Further theoretically oriented research might focus on developing the tools of the TDF (e.g., questionnaires) to more accurately measure barriers and facilitators to the implementation of clinical quality interventions. This might, in turn, make it possible to determine how changeable the TDF domains are (e.g. through mediation analysis, c.f., [71,72]); whether changes in the domains directly influence implementation of clinical quality interventions [73]; and whether targeting domains that represent key barriers and facilitators through matched interventions can improve the implementation of clinical quality interventions. If such an approach proves successful, establishing a model of clinical quality policy implementation using the TDF may well be a possibility.

### Practical implications

There are practical implications of our findings for those wanting to promote the implementation of clinical quality interventions. First, our findings clarify why implementing clinical quality interventions can be so difficult. If only one of the 14 potential barriers to behavior change is present, then this could be enough to engender resistance to uptake by clinicians. This underscores the need for psychologically and socially sophisticated approaches to promoting the implementation of clinical quality interventions, as well as the need to invest significant resources in the implementation phase. Second, it was clear from our results that while clinicians were concerned about the internal and external validity of specific clinical quality instruments, they were not particularly cognizant of, or concerned about, the degree to which general types of interventions were evidence based. This reinforces the well known fact that evidence (or lack thereof) cannot be expected to speak for itself, and that active measures are needed to overcome barriers to the uptake of even the most evidence-based interventions. Finally, because each of the domains of the TDF appeared to be relevant to many different types of clinical quality intervention, it is important that those attempting to implement these interventions begin with an open mind as to which barriers and facilitators are likely to be salient in a particular organizational context. The TDF (in its practical form) is particularly well suited to this kind of “drilling down” because it begins with an exploration of all the possible barriers and facilitators that might exist within a specific local context and then quickly drills down to those that are most salient for the people involved. This approach has already been attempted by those wanting to change behaviours such as hand washing [25], transfusion practices [26] and patient safety guideline implementation [74,75]. In the latter intervention, the authors produced a set of tools for use by those tasked with implementation of guidelines, in order to identify context-specific behaviours to address, barriers to improvement, and strategies to overcome barriers. Our findings provide further support for this approach.

Of course, changes to attitudes and behaviours alone might not be enough, and structural interventions might also be needed. Even where this is the case, however, the TDF would still have an important role in identifying the organizational and structural barriers that are most salient to practitioners in a particular context.

### Strengths, limitations and future directions

We have achieved a synthesis of the studies we reviewed, in that we developed concepts from our data and then demonstrated their relevance to the TDF, rather than simply collating the results of other studies under preexisting headings from the outset. We have identified commonalities and made an otherwise inaccessible literature available to health care practitioners and policy makers, and we have demonstrated the relevance of the TDF to clinical quality interventions.

Our approach has limitations. By necessity, we needed to sacrifice fine detail embedded in some of the individual qualitative studies. This might be viewed as a weakness by those who believe that the main value of qualitative research lies in the detail of individual studies [76] or in the detailed synthesis of a small number of studies. In addition, we were unable to distinguish clearly between clinicians’ perspectives and qualitative researchers’ interpretations of these perspectives. It would be useful to understand the degree to which the domains of the TDF were first- or second-order constructs in qualitative research, but that is not possible to uncover in a study of this kind. Moreover, because we used existing research, we were unable to ask clinicians focused questions about the TDF. Further research would usefully focus specifically on the TDF rather than on barriers and facilitators in general. Finally, further research might usefully systematically compare attitudes towards interventions that have a strong evidence base, against attitudes towards non evidence-based interventions.

The TDF framework itself also has limitations. It does not specify relationships between each of the determinant areas. We can follow the logic, for example, from the Theory of Planned Behaviour [20], that intention is directly related to behaviour, but that the relationships between behaviour and other constructs in the model are mediated partly or fully through intention. By contrast, such patterns cannot be elicited by using the TDF. This can make it difficult to determine the origin of a facilitator or a barrier. For instance, for a specific clinical behaviour, it might be that low ‘beliefs about capabilities’ are a result of a lack of training for adequate skills – as such, ‘skills’ may be the barrier to address first. Alternatively, it may be that low ‘beliefs about capabilities’ are related to a past negative experience, and therefore it would be appropriate to design interventions to target this domain. Thus, in addition to using questionnaire-based measures to identify barriers and facilitators, it may be necessary to further clarify the relationships between each of the determinant areas through, for example, focus group discussions. Related to this is the reality that the domains of the TDF are not mutually exclusive. This is not, however, a problem—and may be a strength—because the tool is designed primarily for practical use rather than as a research tool where definitions need to be precise and mutually exclusive.

## Conclusions

The TDF maps to the barriers and facilitators to uptake of clinical quality interventions. This framework appears to provide a comprehensive account of the barriers to, and facilitators of, uptake of even the most “evidence-based” clinical quality interventions. Notwithstanding the limitations of our study, we believe that the TDF can usefully be used by those wanting to develop strategies to address specific barriers and levers to the implementation of a range of clinical quality interventions.

## Endnote

^a^We chose to use the phrase “clinical governance” in our search because this is a term that is commonly used in Australia, the UK, New Zealand and Canada to describe clinical quality initiatives. Our intention was to use other phrases to capture further articles, but this was not necessary as the initial search elicited a sufficient number of articles.

## Competing interests

The authors declare that they have no competing interests.

## Authors’ contributions

WL conducted the literature review and the initial coding of the qualitative studies. All authors synthesized the codes into categories and “mapped” these codes/categories to the domains of the TDF. All authors contributed to the writing and editing of the article. All authors read and approved the final manuscript.

## Pre-publication history

The pre-publication history for this paper can be accessed here:

http://www.biomedcentral.com/1472-6963/13/530/prepub

## Supplementary Material

Additional file 1 **Table S1.** Details of included studies. Click here for file
