# Supplementary material for: Can the theoretical domains framework account for the implementation of clinical quality interventions?
Source: BMC Health Serv Res. 2013 Dec 21;13:530. doi: 10.1186/1472-6963-13-530 (PMC3901331; doi:10.1186/1472-6963-13-530)
Supplement: Additional file 1 — Table S1. Details of included studies. [file 1472-6963-13-530-S1.doc]

**Additional file Table S1. Details of included studies**

| **Citation** | **Group** | **Country** | **N** | **Clinical governance type** | **Intervention detail** | **Method used to elicit perceptions** |
| --- | --- | --- | --- | --- | --- | --- |
| Andersen (2002). Implementing a new drug record system: a qualitative study of difficulties perceived by physicians and nurses.  Qual Saf Health Care. 2002, 11(1):19-24 | Physicians and nurses | Denmark | 15 | Risk management | A new drug prescribing system | Qualitative interviews |
| Black, & Thompson (1993). Obstacles to medical audit: British doctors speak. Soc Sci Med. 1993, 36(7):849-56. | Consultants and junior doctors | United Kingdom | 62 | Audit | Medical audit in general, geriatric, and A&E departments | Qualitative interviews |
| Bowie, McKay, Dalgetty, & Lough (2005). A qualitative study of why general practitioners may participate in significant event analysis and educational peer assessment. Qual Saf Health Care. 2005, 14(3):185-9. | General practitioners | United Kingdom | 21 | Risk management | Serious event analysis (SEA) and peer assessment | Qualitative focus group interviews |
| Braithwaite, et al. (2005). A tale of two hospitals: assessing cultural landscapes and compositions. Soc Sci Med. 2005, 60(5):1149-62. | Medical, nursing and administrative staff | Australia | 50 (interviews)  + 68 (focus groups) | Structural | Organisational change from traditional clinical structures to clinical directorate | Qualitative interviews and focus groups (plus extensive ethnographic observation) |
| Brown, Crawford, & Darongkamas (2000). Blurred roles and permeable boundaries: the experience of multidisciplinary working in community mental health.  Health Soc Care Community. 2000, 8(6):425-435. | Multi-disciplinary teams including community mental health professionals, occupational therapists, clinical psychologists, psychiatrists, and mental health support workers | United Kingdom | 29 | Structural | Change from single- to multi-disciplinary teams | Qualitative interviews |
| Brown & Crawford (2003). The clinical governance of the soul: ‘deep management’ and the self-regulating subject in integrated community mental health teams. Soc Sci Med. 2003, 56(1):67-81. | Multi-disciplinary teams including community mental health professionals, occupational therapists, clinical psychologists, psychiatrists, and mental health support workers | United Kingdom | 29 | Structural | Change from single- to multi-disciplinary teams | Qualitative interviews |
| Brown, Crawford, Nerlich, & Koteyko (2008).The habitus of hygiene: discourses of cleanliness and infection control in nursing work. Soc Sci Med. 2008, 67(7):1047-55 | Nurses (matrons), infection control staff, operating theatre staff | United Kingdom | 22 | Risk management | Various practices encouraged by national and organisational policy in relation to healthcare acquired infection | Qualitative interviews |
| Brownlie & Howson (2006). Between the demands of truth and government’: health practitioners, trust and immunisation work. Soc Sci Med. 2006, 62(2):433-43 | Health visitors, practice nurses, general practitioners, | United Kingdom | S1 = 37  S2 = 21 | Audit | Practice target setting for an immunisation programme | Qualitative interviews, telephone interviews, focus groups. (Secondary analysis of data from two separate but related studies). |
| Campbell et al (2002). Implementing clinical governance in English primary care groups/trusts: reconciling quality improvement and quality assurance. Qual Saf Health Care. 2002,11(1):9-14. | Chief executives, clinical governance  leads, mental health leads, lay board members, board chairs, executive  committee lead | United Kingdom | 50 | General | Primary care groups and Trusts set up in 1998 and charged with implementing clinical governance over 2 years | Qualitative case studies using semi-structured interviews and case note review |
| Carlsen, Glenton, & Pope (2007). Thou shalt versus thou shalt not: a meta-synthesis of GPs’ attitudes to clinical practice guidelines.  Br J Gen Pract. 2007, 57(545):971-8. | Meta-synthesis of 12 studies | Norway | 12 studies | Evidence based medicine | Clinical practice guidelines | Meta-synthesis of 12 studies |
| Carlsen & Norheim (2008) “What lies beneath it all?" – an interview study of GPs' attitudes to the use of guidelines. BMC Health Serv Res. 2008, 22;8:218. | General practitioners | Norway | 27 | Evidence based medicine | Clinical practice guidelines | Semi-structured group interviews |
| Cartmill, Soklaridis, & Cassidy (2011). Transdisciplinary teamwork: the experience of clinicians at a functional restoration program. J Occup Rehabil. 2011, 21(1):1-8. | Physiotherapists, occupational therapists, kinesiologists cognitive behavioural therapists, psychologists, return to work employees, resource  Specialty employees and, and customer service employees. | Canada | 10 | Structural | A transdisciplinary model of care in a functional restoration program | Qualitative interviews |
| Curnock et al. (2012). Barriers and attitudes influencing nonengagement in a peer feedback model to inform evidence for GP appraisal. BMC Med Educ. 2012, 23;12:15. | General practitioners | United Kingdom | 13 | Audit | External peer review as part of practice appraisal | Semi-structured interviews |
| Deshpande et al. (2003). Incorporating the views of obstetric clinicians in implementing evidence-supported labour and delivery suite ward rounds: a case study. Health Info Libr J. 2003, 20(2):86-94. | Obstetricians, medical trainees, midwives | United Kingdom | 15 | Evidence based medicine | Evidence-supported delivery  suite ward round | Group and one to one interviews |
| Devine et al. (2010). Prescriber and staff perceptions of an electronic prescribing system in primary care: a qualitative assessment. BMC Med Inform Decis Mak. 2010, 19;10:72. | Primary care physicians and staff | United States | 70 | Risk Management | An electronic prescribing system | Focus group interviews using semi-structured questionnaire |
| Embi et al. (2004).  Impacts of computerized physician documentation in a teaching hospital: perceptions of faculty and resident physicians. J Am Med Inform Assoc. 2004, 11(4):300-9. | Faculty and resident physicians | United States | 20 | Risk Management | A computerised physician documentation system | Semi-structured and group interviews |
| Exworthy et al. (2003). The role of performance indicators in changing the autonomy of the general practice profession in the UK. Soc Sci Med. 2003,56(7):1493-504. | General practitioners | United Kingdom | 29 | Audit | General practice performance indicators | Qualitative interviews |
| Freeman et al. (2009). Health professionals’ enactment of their accountability obligations: doing the best they can. Soc Sci Med. 2009, 69(7):1063-71 | Occupational therapists | Canada | 21 | General | Accountability obligations within the current practice context | Qualitative interviews |
| Gagnon et all. (2012). Systematic review of factors influencing the adoption of information and communication technologies by healthcare professionals. J Med Syst. 2012, 36(1):241-77. | Systematic review | Multiple | 49 studies | Risk management | Information and communication technologies | 49 studies used a qualitative approach |
| Georgiou et al. (2009). Computerized Provider Order Entry—What are health professionals concerned about? a qualitative study in an Australian hospital. Int J Med Inform. 2009, 78(1):60-70. | Doctors, pharmacists, nurses, managers, senior health executives | Australia | 50 | Risk management | Computerised medicines management system | Semi-structured interviews and focus groups |
| Goodwin & Happell (2007).  Goodwin & Happell (2008). Psychiatric nurses' attitudes toward consumer and carer participation in care (Parts 1 and 2). Policy Polit Nurs Pract. 2007, 8(4):276-84; Policy Polit Nurs Pract. 2008, 9(4):249-56. | Psychiatric nurses | Australia | 30 | Structural | A policy on consumer a and carer participation in mental health delivery | Focus group interviews |
| Greenhalgh & Douglas (1999).  Experiences of general practitioners and practice nurses of training courses in evidence based health care: a qualitative study.  Br J Gen Pract. 1999, 49, 536-540 | General practitioners and practice nurses | United Kingdom | 30 | EBM | Training course in evidence-based health care | Interviews |
| Harrison & Dowswell (2002). Autonomy and bureaucratic accountability in primary care: what English general practitioners say. Sociol Health Illn. 2002, 24(2), 208-226. | General medical practitioners | United Kingdom | 49 | General | Policies requiring clinical accountability | Semi-structured interviews |
| Hogan et al. (2007). Consultants’ attitudes to clinical governance:  barriers and incentives to engagement. Public Health. 2007, 121(8):614-22 | Medical specialists |  | 24 | General | Perceptions of clinical governance in their hospital | Semi-structured interviews |
| Holmström (2007). Decision aid software programs in telenursing: not used as intended? Experiences of Swedish telenurses. Nurs Health Sci. 2007, 9(1):23-8. | Telenurses | Sweden | 12 | Evidence based medicine | Decision aid software programs for telenursing | Qualitative interviews |
| Hudelson et al (2008).  What is quality and how is it achieved? Practitioners’ views versus quality models. Qual Saf Health Care. 2008, 17(1):31-6. | Hospital-based doctors and nurses | Switzerland | 21 | General | Quality initiatives in general | Semi-structured interviews |
| Johnston et al. (2000).  Reviewing audit: barriers and facilitating factors for effective clinical audit. Qual Health Care. 2000, 9(1):23-36. | Multiple (literature review) | Multiple countries | 93 studies (some qualitative) | Audit | Clinical/medical audit | Multiple including qualitative (literature review) |
| Johnston et al (2011).  Why are some evidence-based care recommendations in chronic obstructive pulmonary disease better implemented than others? Perspectives of medical practitioners. Int J Chron Obstruct Pulmon Dis. 2011, 659-67. | Hospital and primary care clinicians | Australia | 16 | Evidence-based medicine | Clinical practice guidelines | Semi-structured interviews |
| Kendall et al. (2009).  When guidelines need guidance: considerations and strategies for improving the adoption of chronic disease evidence by general practitioners. J Eval Clin Pract. 2009, 15(6):1082-90. | General practitioners | Multiple countries | Literature review of multiple studies | Evidence-based medicine | Clinical practice guidelines | Multiple including qualitative (literature review) |
| MacFarlane et al. (2004).  RCGP Quality Team Development Programme: an illuminative evaluation. Qual Saf Health Care. 2004, 13(5):356-62. | General practitioners and other general practice staff, quality assessors and managers | United Kingdom | 34 | Structural | A quality team development programme | Semi-structured interviews |
| Marshall et al. (2002) A qualitative study of the cultural changes in primary care organisations needed to implement clinical governance. Br J Gen Pract. 2002, 52(481):641-5. | Senior primary care managers and clinicians | United Kingdom | 50 | General | Clinical governance in general | Semi-structured interviews and document analysis |
| Marshall, Hiscock & Sibbald (2002b) Attitudes to the public release of comparative information on the quality of general practice care: qualitative study. BMJ. 2002, 325(7375):1278. | General practitioners, clinical governance leads, service users | United Kingdom | 77 (12 focus groups) | Audit | Public release of comparative information on the quality of general practice care | Focus groups |
| McKay et al. (2008).  Acceptability and educational impact of a peer feedback model for significant event analysis. Med Educ. 2008, 42(12):1210-7 | General practitioners | United Kingdom | 9 | Risk management | Independent, external review of significant event analysis by trained peers | Semi-structured interviews |
| Parker et al. (2007).  Balancing participation and expertise: a comparison of locally and centrally  managed health care quality improvement within primary care practices. Qual Health Res. 2007, 17(9):1268-79. | Primary care doctors and other practice staff | United States | 4 facilities | Structural | Locally vs. centrally managed quality programs | Field notes, semi-structured interviews, |
| Pullon, McKinlay, Dew (2009). Primary health care in New Zealand: the impact of organisational factors on teamwork. Br J Gen Pract. 2009, 59(560):191-7. | Primary care doctors and nurses | New Zealand | 18 | Structural | Interprofessional relationships, teamwork, and collaborative patient care | In-depth interviews |
| Rashidian, Eccles, Russell. (2008). Falling on stony ground? A qualitative study of implementation of clinical guidelines’ prescribing recommendations in primary care. Health Policy. 2008, 85(2):148-61. | Academics in primary care and non-academic general practitioners | United Kingdom | 25 | Evidence-based medicine | Prescribing clinical guidelines | Interviews |
| Reeves & Lewin (2004). Interprofessional collaboration in the hospital: strategies and meanings. J Health Serv Res Policy. 2004, 9(4):218-25. | Doctors, nurses, therapists, social workers | United Kingdom | 49 | Structural | Interprofessional collaboration | Individual and group interviews, observations |
| Richard, Rawal & Martin (2005). An ethical framework for cardiac report cards: a qualitative study. BMC Med Ethics. 2005, 6:E3. | Cardiac patients, administrators, cardiac nurses, cardiologists, other | Canada | 12 | Audit | Cardiac “report cards” | Delphi technique |
| Rousseau et al. (2003).  Practice based, longitudinal, qualitative interview study of computerised evidence based guidelines in primary care. BMJ. 2003, 326(7384):314. | General practitioners, practice managers, nurses | United Kingdom | 13 | Evidence-based medicine | Computerised evidence based guidelines in primary care | Longitudinal interviews |
| Rowan et al. (2006).  Family physicians’ reactions to performance assessment feedback. Can Fam Physician. 2006, 52(12):1570-1. | Family medicine physicians | Canada | 8 | Audit | Performance assessment feedback | Semi-structured interviews |
| Som (2005). Nothing seems to have changed, nothing seems to be changing and perhaps nothing will change in the NHS: doctors' response to clinical governance. Int J Public Sector Management. 2005. 18(5), 463-477. | Doctors | United Kingdom | 33 | General | Clinical governance in general | Semi-structured interviews |
| Sweeney et al. (2002). Softly, softly, the way forward? A qualitative study of the first year of implementing clinical governance in primary care. Prim Health Care Res Dev. 2002, 3, 53-64. | Primary Care Organisation (PCO) clinical governance leads | United Kingdom | 16 | General | Clinical governance in general | Focus groups, interviews, negotiated feedback reports |
| Tait (2004). Clinical governance in primary care: a literature review. J Clin Nurs. 2004, 13(6):723-30. | Multiple (systematic review) | United Kingdom | Literaure review of multiple studies | General | Clinical governance in general | Multiple including qualitative (literature review) |
| Thomassen et al. (2010). Checklists in the operating room: help or hurdle?  A qualitative study on health workers’ experiences. BMC Health Serv Res. 2010, 10:342. | Nurses and physicians | Norway | 14 | Risk management | Operating room checklists | Focus group interviews |
| Travaglia, Westbrook & Braithwaite (2009). Implementation of a patient safety incident management system as viewed by doctors, nurses and allied health professionals. Health (London). 2009, 13(3):277-96. | Doctors, nurses, allied health | Australia | 2185 (a survey with a free-text section) | Risk management | An electronic incident management system | Free text as part of a survey |
| Tufano, Ralston & Martin (2007). Providers’ experience with an organizational redesign initiative to promote patient-centered access: a qualitative study. J Gen Intern Med. 2008, 23(11):1778-83. | Primary care, medical, and surgical care providers | United States | 22 | Structural | Initiative to promote patient-centred access | Semi-structured interviews |
| Walshe et al. (2001). The external review of quality improvement in health care organizations: a qualitative study. Int J Qual Health Care. 2001, 13(5):367-74. | Clinicians, senior managers, members of a clinical governance review team | United Kingdom | 163 | Audit | Clinical governance review visits | Face-to-face and telephone interviews |
| Watkins et al. (2004). Factors affecting feasibility and acceptability of a practice-based educational intervention to support evidence-based prescribing: a qualitative study. Fam Pract. 2004, 21(6):661-9. | Doctors and facilitators | United Kingdom | 4 practices | Evidence-based medicine | Practice-based educational intervention to support evidence-based prescribing | Observation and interviews |
| Wellard et al. (2003). Consumer participation in acute care settings: an Australian experience. Int J Nurs Pract. 2003, 9(4):255-60. | Nurses | Australia | 20 | Structural | Consumer participation in acute care | Focus group interviews and observation |
| Wilkinson et al. (2000). Reactions to the use of evidence-based performance indicators in primary care: a qualitative study. Qual Health Care. 2000, 9(3):166-74. | General practitioners, practice managers, practice nurses | United Kingdom | 52 | Audit | Performance indicators | Semi-structured interviews |
